# Efficient Synthesis of Unprotected C-5-Aryl/Heteroaryl-2'-deoxyuridine via a Suzuki-Miyaura Reaction in Aqueous Media

**DOI:** 10.3390/molecules171214409

**Published:** 2012-12-05

**Authors:** Nathalie Fresneau, Marie-Aude Hiebel, Luigi A. Agrofoglio, Sabine Berteina-Raboin

**Affiliations:** Institut de Chimie Organique et Analytique, Université d’Orléans, UMR CNRS 7311, BP 6759, 45067 Orléans Cedex 2, France

**Keywords:** unnatural nucleosides, Suzuki-Miyaura coupling, aqueous conditions

## Abstract

Following our previous results on an environmentally benign one-pot Sonogashira**-**cyclization protocol to obtain substituted furopyrimidine nucleosides under aqueous conditions, we investigate herein the Suzuki-Miyaura cross-coupling reactions of aryl and heteroaryl derivatives at the C5 position of unprotected 2'-deoxyuridine in the same media with a common catalyst system avoiding exotic ligands, since palladium acetate and triphenylphosphine afforded the expected products in moderate to good yields.

## 1. Introduction

Nucleosides attract attention due to the central role they play in all living systems. Therefore synthesis of unnatural nucleosides arises continuous interest because of their wide biological potential. For instance, 5-substituted 2'-deoxyuridines have been reported as efficient candidates in DNA labeling, modification, and other studies [1,2,3,4,5,6,7,8,9,10,11,12,13,14,15,16,17], and they also exhibit significant antiviral [18,19,20,21,22,23], antibacterial [24], and anticancer activities [25,26,27]. Due to the importance of modified nucleosides, all major classes of palladium-catalyzed reactions have been extensively developed to introduce various substituents [28,29,30,31,32,33,34,35,36,37,38]. Among them, the Suzuki-Miyaura reaction is a powerful and widely used method for carbon-carbon cross coupling reactions. Until lately, this reaction would be carried out in lipophilic media that required working with protected nucleosides. However protection/deprotection sequences induce generally a loss of material and increase the waste production. Recently, Suzuki-Miyaura reactions on unprotected 2'-deoxyuridines in an aqueous-organic solvent system were described, where tetrahydrofuran, acetonitrile, methanol or dimethylformamide was used as co-solvent [1,2,3,4,5,6,7,8,9,10,11,12,13,14,15,16,17,18,19,20,21,22,23,24,25,26,27,39,40]. To the best of our knowledge, only two examples in the 5-iodouridine [41,42] and one in the 2'-deoxy-uridine [43] series are reported in the literature where the experimental conditions required either tris(3-sulfonatophenyl)phosphine trisodium salt (TPPTS) as a specific ligand or palladium supported on reverse phase glass beads. This induced us to disclose herein a similar straightforward method as a natural extension of the current available methods. Based on our interest in environmentally sound processes [44,45], we investigated the development of a Suzuki-Miyaura reaction with 2'-deoxyuridines in a completely aqueous medium using a readily available and inexpensive catalyst/ligand system.

## 2. Results and Discussion

The conditions of the reaction were optimized using the unprotected 5-iodo-2'-deoxyuridine **1** (5-IdU) and 4-methoxyphenylboronic acid. We started by using a mixture of water and acetonitrile in presence of palladium acetate (3 mol %), triphenylphosphine (5 mol %), and sodium carbonate (1.5 equiv) at 80 °C. After 4 hours under these conditions, the starting material **1** was completely consumed and the expected product was isolated in 62% yield. Then, we were pleased to observe that a complete aqueous medium did not prevent the reaction from proceeding but even slightly improved the yield (Table 1, entry 2). An increase in the catalyst loading induced no noticeable change. However the concentration of the reaction mixture appeared to be significant, since **2b** was obtained in 75% yield (entry 4). Further optimizations showed that increasing the amount of boronic acid or replacing the ligand by tri(4,6-dimethyl-3-sulfonatophenyl)phosphine trisodium (TXTPS) [46,47], CataXCium F. Sulf. [48] or tris[bis(*N*-2-hydroxyethyl)aminomethyl]phosphine [49], which are well-known to be highly hydrophilic, did not improve the reaction outcome (entries 4–7) [50].

**Table 1:** Suzuki-Miyaura cross coupling optimization. 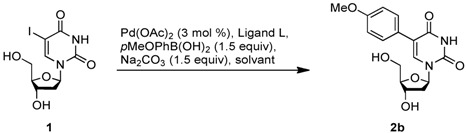

Entry	Ligand (L) ^a^	Solvent	Conditions	Yield (%) ^b^
1	PPh_3_	H_2_O:CH_3_CN 2:1	80 °C, 4 h	62
2	PPh_3_	H_2_O (5 mL)	80 °C, 4 h	67
3	PPh_3_^c^	H_2_O (5 mL)	80 °C, 4 h	69
4	PPh_3_	H_2_O (2.5 mL)	80 °C, 4 h	75 (74) ^d^
5	TXPTS	H_2_O (2.5 mL)	80 °C, 4 h	71
6	CataCXium F sulf	H_2_O (2.5 mL)	80 °C, 4 h	traces
7	P(CH_2_N(C_2_H_4_OH)_2_)_3_	H_2_O (2.5 mL)	80 °C, 4 h	70
8	PPh_3_	H_2_O (2.5 mL)	120 °C, 10 min MW	75 (66) ^e^
9	PPh_3_	H_2_O (2.5 mL)	120 °C, 10 min MW	70 ^f^

^a^ Ratio Pd/L: 1/1.8; ^b^ Isolated yield; ^c^ Pd(OAc)_2_ (10 mol %) and PPh_3_ (25 mol %); ^d^ with 2 equiv. of R-B(OH)_2_; ^e^ with 1 mL H_2_O; ^f^ with Na_2_PdCl_4_.

Then, under the best conditions, the use of microwave irradiation significantly reduced the reaction time with the same yield. Concentrating the media and changing the catalyst induced no positive changes (entries 8 and 9).

To probe the scope of the reaction, the use of different arylboronic acids was examined (Table 2). The expected products were cleanly obtained in good yields with substrates that contained electron withdrawing and donating groups in the *para *and *meta *positions (entries 1 and 2). It is worth noting that the use of potassium trifluoroborate is compatible with these experimental conditions as a strict stoichiometric amount of potassium phenyltrifluoroborate gave **2a** with the same range of yields (entry 1).

**Table 2:** Substrates scope. 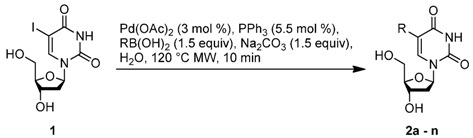

Entry	RB(OH)_2_	Products		Yield (%) ^a^
1	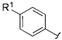	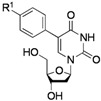	**2a**: R^1^ = H	70 (62) ^b^
			**2b**: R^1^ = OMe	75
			**2c**: R^1^ = Ac	72
			**2d**: R^1^ = CHO	79
			**2e**: R^1^ = F	74
			**2f**: R^1^ = NO_2_	68
2	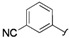	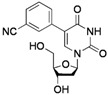	**2g**	70
3	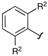	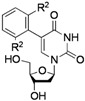	**2h**: R^2^ = Me	-
			**2i**: R^2^ = OMe	-
4	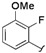	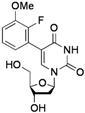	**2j**	53 ^c^
5	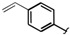	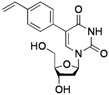	**2k**	30
6	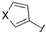	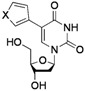	**2l**: X = O	67 (73) ^d^
			**2m**: X = S	81
7	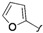	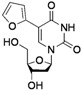	**2n**	44^c^

^a^ Isolated yield; ^b^ with 1 equiv. Ph-BF_3_K; ^c^ with 3 equiv. of R-B(OH)_2_; ^d^ with 2 equiv. of R-B(OH)_2_.

The effects of steric hindrance were also tested with mono- and di-*ortho*-substituted boronic acids showing a limitation of the method by causing a moderate and drastic loss of yield, respectively (entries 3 and 4). Then the challenging styrene-4-boronic acid was also tried to compare the reaction with the competitive Heck cross-coupling. In this case, the desired product was isolated in 30% yield with no trace of the alkenyl compound [51]. To expend the range of applicable substrates, 5-iodo-2'-deoxyuridine **1** was coupled with a variety of heteroarylboronic acids (Table 2, entries 6 and 7). Moderate to good yields were observed with thiophene-3-, furan-2-, and furan-3-boronic acids requiring occasionally a larger excess of the boronic moiety (entries 6 and 7). Unfortunately, with pyridin-2- and pyridin-3-boronic acids no reaction was observed, and the same result was obtained with the more stable potassium pyridin-3-trifluoroborate.

## 3. Experimental

### 3.1. General

Solvents and reagents were purchased from commercial suppliers and used without further purification. ^1^H-NMR and ^13^C-NMR were recorded on a Bruker Avance DPX 250 or 400 MHz spectrometers. High-resolution mass spectra (HRMS) were recorded with a TOF spectrometer in the electrospray ionisation (ESI) mode or with a Finnigan MAT 95 XL in the chemical ionisation (CI) mode at the Regional Center of Physical Measurement University Blaise Pascal. All commercial solvents were used without further purification. Column chromatography was carried out using Silica gel 60N (spherical, neutral, 40–63 µm, Merck). Melting point was measure on Thermo Scientific 9200. Intfrared (IR) spectra were obtained on FT-IR Thermo Scientific Nicolet iS10. Thin layer chromatography (TLC) was carried out on Merck silica gel 60F_254_ precoated plates. Visualization was made with ultraviolet light.

### 3.2. General procedure

Under nitrogen, 5-IdU **1** (100 mg, 0.282 mmol), PPh_3_ (4.1 mg, 0.016 mmol), sodium carbonate (44.8 mg, 0.423 mmol), and ary/hetarylboronic acid (0.423 mmol) were dissolved in water (2.5 mL). Then Pd(OAc)_2_ (2.5 mg, 0.011 mmol) was added to the mixture before sealing the vial. Then the mixture was irradiated for 10 min at 120 °C. After completion, water was added (5 mL) and the pH was adjusted to 7 using aqueous HCl 10%. The solution was concentrated under reduced pressure and the residue was finally purified by silica gel chromatography to afford the desired product.

*5-Phenyl-2'-deoxyuridine* (**2a**). DCM/MeOH (96/4), spectroscopic data conformed to the literature [40].

*5-(4-Methoxyphenyl)-2'-deoxyuridine* (**2b**). DCM/MeOH (96/4), spectroscopic data conformed to the literature [40].

*5-(4-Acetylphenyl)-2'-deoxyuridine* (**2c**). DCM/MeOH (96/4), white solid (72% yield); mp >250 °C (slow degradation); ^1^H-NMR (250 MHz, DMSO-*d_6_*) δ 11.60 (bs, 1H), 8.41 (s, 1H), 7.96 (d, *J* = 8.5 Hz, 2H), 7.75 (d, *J* = 8.5 Hz, 2H), 6.24 (t, 1H, *J* = 6.5 Hz), 5.28 (d, 1H, *J* = 2.7 Hz), 5.19 (t, 1H, *J *= 3.2 Hz), 4.37–4.26 (m, 1H), 3.88–4.81 (m, 1H), 3.69–3.60 (m, 2H), 2.59 (s, 3H), 2.35–2.12 (m, 2H); ^13^C-NMR (125 MHz, DMSO-*d_6_*) δ 197.8, 162.3, 150.2, 139.7, 138.5, 135.6, 128.5, 128.1, 112.6, 88.0, 85.2, 70.5, 61.3, 27.1 (one peak under the DMSO-*d_6_* signal); IR (neat): 3421, 3162, 3054, 2921, 2831, 1671, 1657, 1598, 1558, 958 cm^−1^; HRMS (ESI) calcd. for C_17_H_18_N_2_O_6_Na (M+Na) 369.1063, Found 369.1077.

*5-(4-Formylphenyl)-2'-deoxyuridine* (**2d**). DCM/MeOH (96/4), spectroscopic data conformed to the literature [7].

*5-(4-Fluorophenyl)-2'-deoxyuridine* (**2e**). DCM/MeOH (96/4), spectroscopic data conformed to the literature [40].

*5-(4-Nitrosophenyl)-2'-deoxyuridine* (**2f**). DCM/MeOH (96/4), spectroscopic data conformed to the literature [52].

*5-(3-Cyanophenyl)-2'-deoxyuridine* (**2g**). DCM/MeOH (96/4), white solid (70% yield); mp 179.2–180.5 °C; ^1^H-NMR (250 MHz, DMSO-*d_6_*) δ 11.63 (bs, 1H), 8.34 (s, 1H), 8.01 (s, 1H), 7.89 (d, *J* = 7.9 Hz, 1H), 7.76 (d, *J* = 7.9 Hz, 1H), 7.58 (t, *J* = 7.9 Hz, 1H), 6.22 (t, *J* = 6.5 Hz, 1H), 5.26 (d, *J* = 3.9 Hz, 1H), 5.17 (t, *J* = 4.7 Hz, 1H), 4.35–4.24 (m, 1H), 3.81 (q, *J* = 3.9 Hz, 1H), 3.65 (dd, *J* = 8.6, 4.7 Hz, 1H), 3.58 (dd, *J* = 8.6, 3.9 Hz, 1H), 2.35–2.12 (m, 2H); ^13^C-NMR (125 MHz, DMSO-*d_6_*) δ 162.3, 150.2, 139.7, 135.0, 133.0, 131.6, 131.1, 129.8, 119.3, 111.8, 111.8, 88.0, 85.1, 70.3, 61.2 (one peak under DMSO *d_6_* signal); IR (neat): 3468, 3336, 3207, 3071, 2236, 1682, 1263, 1086, 945, 817 cm^−1^; HRMS (ESI) calcd. for C_16_H_15_N_3_O_5_Na (M+Na) 352.0909, Found 352.0906.

*5-(2-Fluoro-3-methoxyphenyl)-2'-deoxyuridine* (**2j**). DCM/MeOH (96/4), white solid (53% yield); mp 173.2–174.2 °C; ^1^H-NMR (250 MHz, DMSO-*d_6_*) δ 11.52 (bs, 1H), 8.05 (s, 1H), 7.13 (m, 2H), 6.91 (dt, *J* = 6.3, 2.8 Hz, 1H), 6.22 (t, *J* = 6.7 Hz, 1H), 5.25 (d, *J* = 3.6 Hz, 1H), 4.97 (t, *J* = 4.7 Hz, 1H), 4.23–4.25 (m, 1H), 3.84 (s, 3H), 3.81–3.75 (m, 1H), 3.60–3.45 (m, 2H), 2.21–2.12 (m, 2H); ^13^C-NMR (125 MHz, DMSO-*d_6_*) δ 161.8, 150.5, 148.6, 147.7, 140.1, 124.3, 123.1, 121.9, 113.7, 109.3, 88.0, 84.9, 70.8, 61.5, 56.5 (one peak under the DMSO-*d_6_* signal); IR (neat): 3411, 3040, 2943, 2837, 1697, 1663, 1480, 1271, 1096, 1043, 1021, 790 cm^−1^; HRMS (ESI) calcd. for C_16_H_17_N_2_O_6_FNa (M+Na) 375.0968, Found 375.0971.

*5-(4-Vinylphenyl)-2'-deoxyuridine* (**2k**). DCM/MeOH (96/4), pale yellow gel (30% yield); ^1^H-NMR (400 MHz, DMSO-*d_6_*) δ 11.57 (bs, 1H), 8.31 (s, 1H), 7.62 (d, *J* = 8.3 Hz, 2H), 7.53 (d, *J* = 8.3 Hz, 2H), 6.80 (dd, *J* = 17.7, 10.8 Hz, 1H), 6.30 (t, *J* = 6.5 Hz, 1H), 5.91 (d, *J* = 17.7 Hz, 1H), 5.33 (d, *J* = 10.8 Hz, 1H), 5.32 (d, *J* = 3.6 Hz, 1H), 5.19 (t, *J* = 4.8 Hz, 1H), 4.38–4.34 (m, 1H), 3.91–3.82 (m, 1H), 3.71–3.59 (m, 2H), 2.37–2.14 (m, 2H); ^13^C-NMR (125 MHz, DMSO-*d_6_*) δ 162.5, 150.3, 138.4, 136.7, 136.4, 128.4, 126.3, 114.7, 113.4, 88.0, 85.0, 70.6, 61.4 (one peak under the DMSO-*d_6_* signal); IR (neat): 3395, 3056, 2961, 1667, 1262, 1088, 1047, 1027, 792 cm^−1^; HRMS (ESI) calcd. for C_17_H_18_N_2_O_5_Na (M+Na) 353.1113, Found 353.1112.

*5-(Furan-3-yl)-2'-deoxyuridine* (**2l**). DCM/MeOH (96/4), spectroscopic data conformed to the literature [53].

*5-(Thiophen-3-yl)-2'-deoxyuridine* (**2m**). DCM/MeOH (96/4), spectroscopic data conformed to the literature [53].

*5-(Furan-2-yl)-2'-deoxyuridine* (**2n**). DCM/MeOH (96/4), spectroscopic data conformed to the literature [54].

## 4. Conclusions

In summary, we disclose herein a successful Suzuki-Miyaura reaction with 5-iodo-2'-deoxyuridine in a completely aqueous medium. The inexpensive and common triphenylphosphine combined with palladium acetate gave rise to the expected products in moderate to good yields. Furthermore, the variety of aryl and heteroaryl derivatives introduced demonstrates the generality of this method.
